# Tissue Distribution and Associated Toxicological Effects of Decabrominated Diphenyl Ether in Subchronically Exposed Male Rats

**DOI:** 10.5402/2011/989251

**Published:** 2012-01-12

**Authors:** Fuxin Wang, Jianshe Wang, Guocheng Hu, Xiaojun Luo, Bixian Mai, Jiayin Dai

**Affiliations:** ^1^Key Laboratory of Animal Ecology and Conservation Biology, Institute of Zoology, Chinese Academy of Sciences, Beijing 100101, China; ^2^State Key Laboratory of Organic Geochemistry, Guangzhou Institute of Geochemistry, Chinese Academy of Sciences, Guangzhou 510640, China

## Abstract

Concerns about decabrominated diphenyl ether (BDE-209) have arisen recently due to its increasing concentrations in the environment. We investigated the tissue concentration, distribution, and the debromination of BDE-209 after oral exposure, using rats as a model. Three groups of male rats were administrated by oral gavage with corn oil containing 0, 10, or 50 mg/kg bw/day of BDE-209 over 90 days. After exposure, BDE-209 and its metabolites levels in the liver, kidney, and adipose of the rats were measured. The mRNA expression levels of cytochrome P450 (CYP) enzymes in liver, serum thyroid hormone levels, and open-field tests were also measured. BDE-209 and several octa- and nona-BDE congeners were detected in the tissues of the dosed rats, indicating that BDE-209 was bioavailable and biotransformative in male rats. BDE-209 and its debrominated congeners had no mRNA level effect on selective genes from the CYP family in the liver or on the spontaneous behavior of adult male rats. Conversely, the level of thyroid hormone, total triiodothyronine (T_3_) in rats from the dosed treatments increased significantly compared to the control group.

## 1. Introduction

Polybrominated diphenyl ethers (PBDEs) are a class of additive flame-retardant chemicals used in a variety of products, including textiles, plastics, and foam [1]. While PBDEs are beneficial for fire safety and instrumental in saving lives, they have been detected increasingly in the environment, wildlife, and human tissue since their introduction in the 1970s [2].

Decabromodiphenyl ether (BDE-209), a fully brominated PBDE, has its main application in the plastics, electronics, and textile industries [3–5]. Due to its high molecular weight and hydrophobicity, BDE-209 was initially assumed to have little environmental impact during the phases of its use or to be not available biologically. It is blended physically within rather than bounded chemically to polymeric materials. As a result, it can be released from products into the ambient environment during processing and after disposal [6–9]. BDE-209 can be debrominated by photolysis to form octa- and nona-BDEs as initial degradation products [10, 11]. Studies on BDE-209 in exposure studies with fish [12, 13], seals [14], birds [15] and rat [16] have shown that BDE-209 can be debrominated by metabolic routes. An inverse relationship between the potential toxicity and the number of bromine atoms among BDE congeners has been shown in many studies and, generally, lower brominated congeners are more toxic and bioavailable than BDE-209 [17, 18].

Studies have detected BDE-209 in the blood of occupationally exposed and unexposed humans [19, 20]. In addition, recent studies on BDE-209 have also shown it has potential neurotoxic and neonatal risks, causes a decrease in epididymal sperm functions [21], perturbs the thyroid system [1, 22] and hepatic enzyme activity in male mouse offspring [23]. The two U S producers of BDE-209 and the largest U S importer recently announced a BDE-209 phase out by the end of 2013 [24].

In this study, three groups of male Sprague-Dawley (SD) rats were orally administrated with corn oil containing different doses of BDE-209 for 90 days. To investigate their tissue concentration and distribution, BDE-209 levels in the liver, kidney, and adipose were measured after exposure. The mRNA expression levels of cytochrome P450 (CYP) enzymes, thyroid hormone levels, and open-field tests were also measured.

## 2. Materials and Methods

### 2.1. Chemicals

The BDE-209 (≥99% purity) was purchased from Shanghai Jiachen Chemical Co., Ltd. and the corn oil was obtained from Sigma-Aldrich (St. Louis, MO). We purchased PBDE standards, including BDE-28, 47, 66, 99, 100, 138, 153, 154, 183, 196, 197, 202, 203, 206, 207, 208, and 209 from AccuStandard (New Heaven, CT). We obtained ^13^C_12_-PCB-141 and ^13^C_12_-BDE-209 (recovery standards) and ^13^C_12_-PCB-208 (internal standard) from Cambridge Isotope Laboratories Inc. (MA). The recovery standards (BDE-77, 181) and the internal standards (BDE-118, 128) were purchased from AccuStandard. All solvents and reagents used in these experiments were of analytical grade and organic solvents were redistilled using the glass system.

### 2.2. Animals

Male SD rats (21 days old) obtained from Weitong Lihua Experimental Animal Center (Beijing, China) were housed individually and maintained in a mass air-displacement room with a 12-hour light-dark cycle at 20–26°C and a relative humidity of 50–70%. After a one month acclimation period, the rats were separated into three groups (control, 10 and 50 mg/kg/d BDE-209 treatments, *n* = 12 rats per treatment). We selected the liver, kidney, and white adipose of rats to investigate metabolic potentials and products. All rats were sacrificed by cervical dislocation after 90 days of exposure and the open field test. Animal handling protocol was approved by the Institute of Zoology, Chinese Academy of Sciences Institutional Animal Care Committee. Blood was collected from heart after anesthesia using carbon dioxide and centrifuged at 2000 ×g at 4°C for 15 min. Liver, kidney, and white adipose were immediately collected from each rat and tissue samples were frozen in liquid nitrogen and stored at −20°C or −80°C until used for chemical analysis and toxic experiments, respectively.

### 2.3. Sample Extraction and Analysis

Details of the analytical procedures are described elsewhere [25]. Briefly, subsamples (1-2 g) were spiked with recovery standards (^13^C_12_-PCB-141, BDE-77, BDE-181, and ^13^C_12_-BDE-209) and Soxhlet extracted with 1 : 1 hexane :acetone (V/V) for 48 h. We determined lipid content on an aliquot of extract gravimetrically. The remaining extract was further purified with gel permeation chromatography and a 2 g silica gel solid-phase extraction column (Isolute, International Sorbent Technology, UK). The PBDEs were eluted from the silica column with 30 mL of 15 : 15 hexane/dichloromethane. The extract was further concentrated to 200 *μ*L and spiked with internal standards (BDE-118, BDE-128, and ^13^C_12_-PCB-208).

We analyzed samples with a Shimadzu model 2010 gas chromatograph mass spectrometer (GC/MS) (Shimadzu, Japan) under electron capture negative ionization (ECNI) in the selected ion monitoring (SIM) mode using a DB-5HT capillary column (15 m length, 0.25 mm diameter, 0.10 *μ*m thickness). The instrumental conditions are presented elsewhere [25]. Ion fragments m/z 79 and 81 ([Br]^−^) were monitored for PBDEs and their possible degradation/metabolic products. For BDE-209 and ^13^C_12_-BDE209, m/z 486.7, 488.7 and m/z 494.6, 496.6 were respectively recorded. Fragments monitored for surrogate ^13^C_12_-PCB-141 and internal standard ^13^C_12_-PCB208 were m/z 372, 374, and 376, and 474, 476, and 478, respectively.

### 2.4. QA/QC and Data Analysis

Procedural blanks covering the whole process were run in parallel with the samples for each batch of extraction. Blind triplicate samples, triplicate spiked blanks, and triplicate spiked matrices were performed throughout the study. While BDE-209 was found in the procedural blanks, levels were significantly (*P* < 0.05) lower than the levels in the control rat samples. The BDE-209 concentrations in samples were corrected from background concentrations of BDE-209 by subtracting three times the mean BDE-209 level in the blanks. The relative standard deviations (RSD) among triplicate samples were, on average, 3–9.6% for all targets. Retrieval of recovery standards averaged from 76 to 120% in all samples. The limit of detection (LOD) was established as mean values plus three times the standard deviation of the procedural blanks for the congeners detected in the procedural blanks. For congeners which were not detected in the blanks, LOD was defined as the instrumental limit of quantification (signal/noise = 5). Typical LODs ranged from 0.0023 to 0.0923 ng/g lw for tri- to hepta-BDEs, from 0.0001 to 4.3413 ng/g lw for octa- to nona-BDEs, and from 0.0179 to 2.1516 ng/g lw for BDE-209, depending on the sample size. Statistical analysis was performed using SPSS 16.0 for windows (SPSS Inc., Chicago, IL) for comparisons of means between and within treatment groups. Significance was set at *P* < 0.05. Instrumental QC included regular injections of solvent and standard solutions.

### 2.5. Gene Expression Analysis

Total RNA from individual liver samples was isolated by TRIzol reagent (Invitrogen Corp., Carlsbad, CA) in accordance with manufacturer's instructions. The concentration of RNA was measured by absorbance at 260 nm using a UV1240 spectrophotometer (Shimadzu, Japan), and 260/280 nm absorbance ratio for estimating its purity. The cDNA was prepared with a reverse transcription system, which was performed using oligo-(dT)15 primer (Promega, Madison, WI) and M-MuLV reverse transcriptase (Promega, Madison, WI) according to the manufacturer's instructions.

A Stratagene Mx3000P real-time PCR (polymerase chain reaction) apparatus (Stratagene, Cedar Creek, USA) was used to measure real-time PCR amplification and detection by SYBR Green I technology. Each 25 *μ*L of reaction mixture was composed of 12. 5 *μ*L of SYBP Premix Ex Taq with ROX II dye (TAKARA, Dalian, China), forward and reverse primers (10 *μ*M, 0.5 *μ*L each), the cDNA sample (1 *μ*L), and 10.5 *μ*L of nuclease-free water. For data analysis, *β*-actin was used as a housekeeping gene. The PCR amplification protocol was as follows: 95°C for 2 min followed by 40 cycles at 94°C for 5 s, 56°C for *β*-actin (54°C for CYP1A2, 53°C for CYP2B1, 56°C for CYP2B2, 50°C for CYP2C6, 47°C for CYP3A2) for 15 s, and 72°C for 10 s. The expression levels of selected genes and their PCR primers are given in Table 1. Every sample was analyzed in triplicate. The quantification of the transcripts was performed by the 2^−ΔΔCT^ method [26].

### 2.6. Thyroid Hormone Analysis

We measured the serum levels of thyroid hormones, total triiodothyronine (T_3_) and total thyroxine (T_4_), by radioimmunoassay. Blood was collected from all rat groups in the morning of the 90th day to avoid the fluctuation of thyroid hormone levels. Differences between the control and treatment groups were determined using a one-way analysis of variance (ANOVA). The calculation was based on discrimination significance between classes at the level of *P* < 0.05.

### 2.7. Open Field Test

At the end of 90-days exposure, rats were placed in one corner of a square arena (75 cm × 45 cm × 30 cm Plexiglas box, divided into 15 equally sized grids at the bottom) and allowed to explore for 3 minutes. Standard parameters were investigated, including latency, periphery grids, center grids, rearing number, and grooming time. Silence and no direct light were requested during the entire test. The open field apparatus was washed with 75% ethanol before each test to eliminate possible odor clues left by previous subjects.

## 3. Results and Discussion

No mortalities were observed throughout the duration of the study. Whole-body growth rates were significantly lower in both dosed treatment groups (10 mg/kg/d group: 0.59 ± 0.12%; 50 mg/kg/d group: 0.76 ± 0.07%) compared to the control group (1.57 ± 0.05%) (*P* < 0.01), suggesting that BDE-209 had a negative effect on the growth of male rats (Table 2). While absolute and relative liver weights are useful indicators of rat health, no significant variation between the dosed and untreated rats (*P* > 0.05) was observed. In addition, no statistically significant differences in absolute and relative kidney weight were observed between any of the groups (*P* > 0.05).

### 3.1. Tissue Distribution of BDEs in Rats

**Figure 1:**
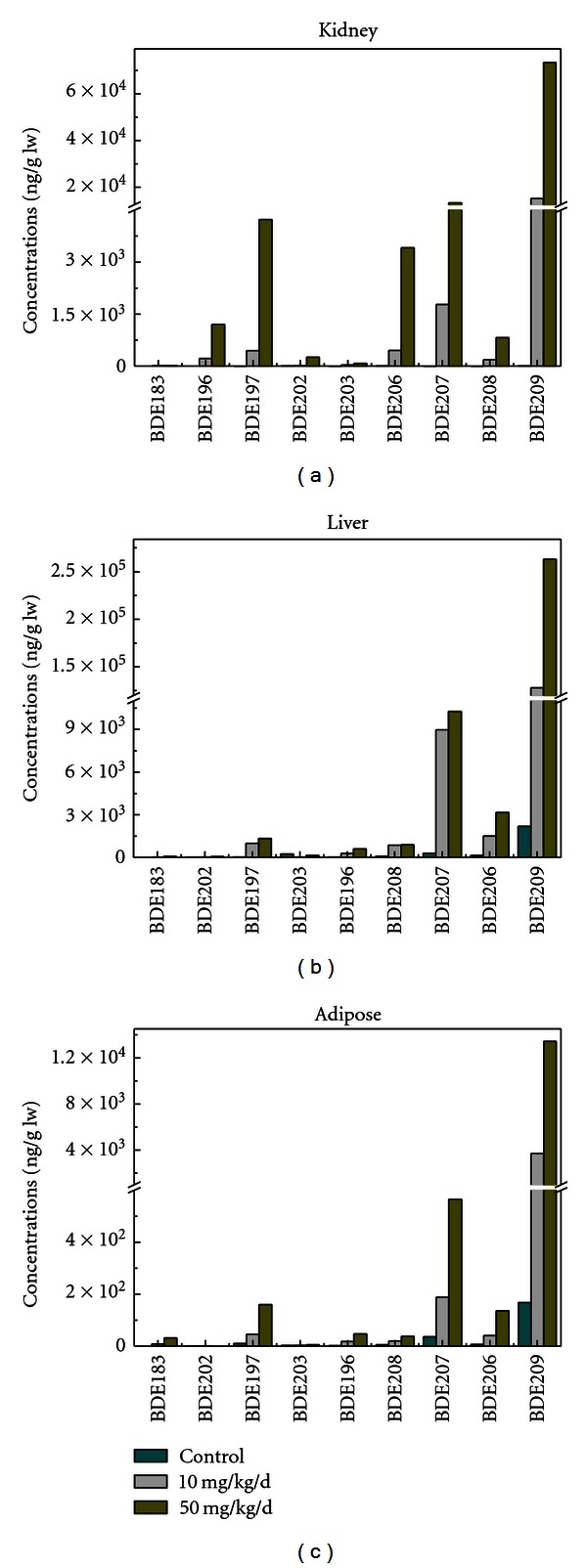
Tissue distribution of BDEs measured in kidney (a), liver (b), and adipose (c) of male rats exposed to BDE-209 for 90 days.

Nine BDE congeners including BDE-183, 196, 197, 202, 203, 206, 207, 208, and 209 were detected in male rats with significantly elevated concentrations in the tissues of dosed rats compared to the control group (Figure 1). Generally, BDEs accumulated in tissues in the order, liver > kidney > adipose, and increased with dose level. The highest concentration was observed for BDE-209 in the two treatments. In the 50 mg/kg/d exposed group, the concentrations of BDE-209 were 73,471, 263,003, and 13,436 ng/g lw in kidney, liver, and adipose tissues, respectively. These levels were 2.05, 4.87, and 3.64 fold higher than that in 10 mg/kg/d group, respectively. Among the eight nonfully brominated BDE congeners (BDE-183, 196, 197, 202, 203, 206, 207, and 208), BDE-207 was predominant in the two treatments, contributing to 57.6%, 70.9%, and 57% of total lower brominated BDEs in the kidney, liver, and adipose tissues of the higher dosed group, respectively. Of next predominance were BDE-197, 206, 196, and 208, which all belong to nona- or octa-BDEs. All these BDEs in the 50 mg/kg/d dosed group accumulated to a higher level than in the 10 mg/kg/d group.

Non-fully brominated BDEs were observed in all rats exposed to BDE-209 treatment. The original source of these low brominated BDEs were thought to have degraded from BDE-209. Other studies have demonstrated that BDE-209 is reductively debrominated to lower brominated congeners in dosed fish and rats, with the major debrominated metabolites being octa- and nona-BDEs [15, 27, 28]. In our study, the same results were obtained in BDE-209 exposed rats where BDE-207 and 197 were the major congeners in the nona- and octa-homologue groups, which could be explained by the selectively removal of bromine atoms from the meta-positions by the catalytic metabolism of deiodinase enzymes within rat tissues [29].

BDE-209 is the most important PBDE detected in environments in China, accounting for 50–90% of total PBDE in sediment and air in typical regions of South China [30]. This study shows that BDE-209 not only accumulated in exposed rats, but it was also a likely source of more hazardous lower brominated BDEs [18, 19]. These results contribute further understanding of the environmental degradation of BDE-209 and toxic potentials of the parent BDE-209 and its debrominated congeners in Chinese environment.

### 3.2. The mRNA Expression Levels of CYP Enzymes

**Figure 2:**
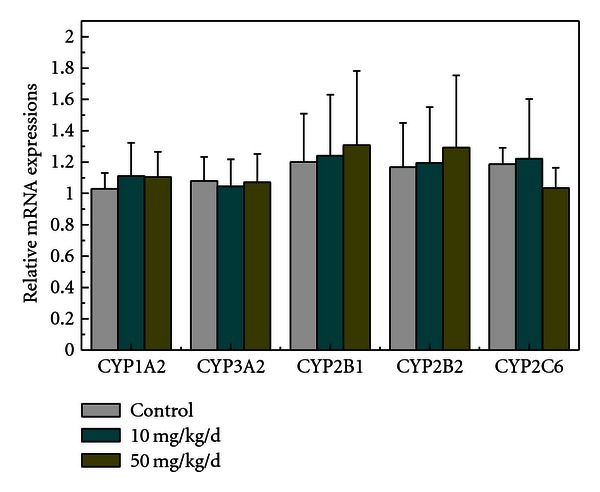
Relative liver mRNA expression of CYP1A2, 3A2, 2B1, 2B2, and 2C6 from control and BDE-209-exposed rats (mean ± SEM; *n* = 6).

The effects of BDE-209 exposure on mRNA gene expression involved in CYPs, proteins responsible for drug oxidation, environmental pollution, and endogenous compounds [31], were determined by real-time PCR (Figure 2). As our results showed that the liver held BDEs to the highest level, the liver was thus selected as the source tissue of these genes. Compared to the control group, no significant changes occurred in the relative mRNA expression of these selected genes in the 10 mg/kg/d or 50 mg/kg/d treatment groups (*P* > 0.05). This suggests that BDE-209 and its debrominated BDEs had no effect on these genes at the mRNA level.

### 3.3. The Thyroid Hormone Levels

To investigate the potential influence of BDE-209 and its debrominated BDEs on endocrine-related processes, thyroid hormone levels in serum were measured by radioimmunoassay (Table 3). Compared to the control group, T_4_ levels in the two treatments tended to be higher, although neither was statistical significantly (*P* > 0.05). For T_3_ levels, however, significant increases were observed in both the 10 mg/kg/d group (*P* < 0.01) and 50 mg/kg/d group (*P* < 0.05). This indicates that BDE-209 altered thyroid hormone homeostasis of male rats to a different degree depending on dosage. In toxicological studies, chemical exposure is supposed to be much higher than that experienced in the environment. In the present paper, the T_3_ level was more significantly increased in 10 mg/kg/d dosed group than those in 50 mg/kg/d dosed group, while concentrations of BDE-209 and the lower brominated BDEs in tissues were much lower in lower dose group than in higher dose group, indicating that the thyroid hormone homeostasis of some organism may be perturbed in the environment because of the accumulation of BDE-209 and/or its debrominated homologous compounds. Multiple animal model studies have demonstrated that PBDEs can perturb the thyroid system, as well as in some in vitro test systems, however, mainly on the T_4_ levels which is not consistent with the present study [1, 32–34]. Thus, further studies are required to determine this different effect and its importance from a toxicological point of view.

### 3.4. Open-Field Tests

**Figure 3:**
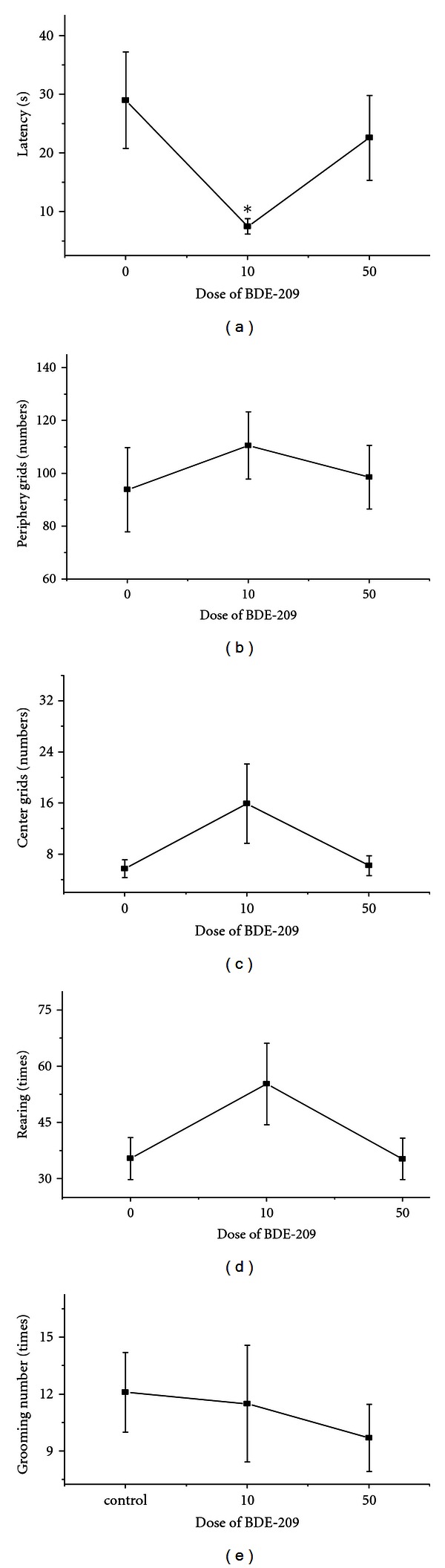
Behavioral responses in open field test of male rats exposed to 10 mg/kg/d or 50 mg/kg/d BDE-209 for 90 days (mean ± SEM; *n* = 6). (a) latency (second); (b) periphery grids (numbers); (c) center grids (numbers); (d) rearing (times); (e) grooming number (times).

Figure 3 presents the open-field tests of male rats exposed to BDE-209 for 90 days. Compared to the control group, the latency of the lower dosed group was significantly shorter (*P* < 0.05). However, no significant change occurred in the number of periphery grids and center grids, or times of rearing and grooming number (*P* > 0.05). None of these parameters was changed for all of the parameters in the higher dosed group.

In Viberg's study, neurobehavioral derangements were observed in adult mice receiving BDE-209 during a defined period of neonatal brain development [35]. Rice also found that neonatal exposure to BDE-209 caused dose-response changes in spontaneous behavior and cholinergic susceptibility in adult mice [36]. Developmental delays and locomotor activity in the C57BL6/J mouse following neonatal exposure to BDE-209 were observed. The open-field test is commonly used to investigate spontaneous behavior in animals. Results from the present study suggest that spontaneous behavior in dosed rats did not change significantly compared to the control group. The probable reason is that the nervous systems in charge of spontaneous behavior in the 50-day-old rats were already mature when BDE-209 exposure began. This suggests that BDE-209 may not affect spontaneous behavior in adult male rats.

## 4. Conclusion

The present study demonstrated that BDE-209 was bioavailable and biotransformative in adult male rats exposed to 10 mg/kg/d or 50 mg/kg/d dosed treatments, although the concentration of BDEs in tissues was significantly lower in the 10 mg/kg/d group. It was also clear that individual BDEs exhibited different tissue distribution; although retention in the liver was the highest. Both BDE-209 and its debrominated BDEs had no effect on the mRNA level of selective CYP family genes from the liver. In addition, the spontaneous behavior of adult male rats did not change after subchronic exposure to BDE-209 over 90 days. Although total T_3_ level increased significantly after exposure, it showed a different pattern to previous studies in which PBDEs perturbed T_4_ levels. Further investigation is warranted to determine the mechanism for disturbance of thyroid system.

## Figures and Tables

**Table 1:** Sequences of primers used for real-time RT-PCR amplification.

Target gene	GeneBank accession no.^a^	5′-3′ Primer sequences^b^	Product length (bp)	Tm (°C)
*β*-actin	NM_031144	FW: TCGTGCGTGACATTAAAGAG	134	56
		RW: ATTGCCGATAGTGATGACCT		
CYP1A2	NM_000761	FW: GTGGAATCGGTGGCTAAT	105	54
		RW: CACAAAGTCCTTGCTGCTC		
CYP2B1	NM_001134844	FW: GCCTCCTCAATTCCTTCA	99	53
		RW: TGTCTGTCCCACATAGCAT		
CYP2B2	XM_001062335	FW: AGGAGAAGTCGAACCACCAC	82	56
		RW: GAGCAGGAAACCATAGCG		
CYP2C6	XM_001066767	FW: TGTAGAGTTTCAGGGATGG	94	50
		RW: AGCAGTGAGATTGGGAAG		
CYP3A2	NM_153312	FW: GTCTCATAAAGCCCTGTC	81	47
		RW: CTGCTGGTGGTTTCATAG		

^a^GeneBank accession number used to design the primers.

^b^FW: forward primer; RW: reverse primer.

**Table 2:** Whole body growth rates, and absolute and relative liver and kidney weight of the control rats and rats exposed to 10 or 50 mg/kg/d BDE-209 for 90 days.

	Control	BDE-209 (10 mg/kg/d)	BDE-209 (50 mg/kg/d)
Whole-body growth rates (%)^a^	1.57 ± 0.05	0.59 ± 0.12**	0.76 ± 0.07**
Absolute liver weight (g)	13.69 ± 0.10	15.32 ± 0.84	14.38 ± 0.28
Absolute kidney weight (g)	3.24 ± 0.15	3.39 ± 0.14	3.33 ± 0.04
Relative liver weight (%)^b^	2.98 ± 0.05	3.06 ± 0.15	2.85 ± 0.07
Relative kidney weight (%)^b^	0.70 ± 0.03	0.68 ± 0.01	0.66 ± 0.03

Data are represented as mean ± SEM from six rats per group. ^a^The average growth rate after 90 days. ^b^Percentage of total body weight. Statistically significant differences between the controls and treatments are indicated by ** for *P* < 0.01.

**Table 3:** Effects of BDE-209 on serum thyroid hormones.

		BDE-209 (mg/kg/d)	
	Control (corn oil)	10	50
T_3_ (ng/mL)	0.53 ± 0.07	0.84 ± 0.05**	0.62 ± 0.06*
T_4_ (ng/mL)	71.73 ± 5.37	74.06 ± 3.08	79.71 ± 3.23

Data are represented as mean ± SEM from six rats per group. Statistically significant differences between the control and treatment groups are indicated by *for *P* < 0.05, and **for *P* < 0.01.
